# Regional variation in hip and knee arthroplasty rates in Switzerland: A population-based small area analysis

**DOI:** 10.1371/journal.pone.0238287

**Published:** 2020-09-21

**Authors:** Maria M. Wertli, Judith M. Schlapbach, Alan G. Haynes, Claudia Scheuter, Sabrina N. Jegerlehner, Radoslaw Panczak, Arnaud Chiolero, Nicolas Rodondi, Drahomir Aujesky

**Affiliations:** 1 Department of General Internal Medicine, Bern University Hospital, Inselspital, University of Bern, Bern, Switzerland; 2 Institute of Social and Preventive Medicine, University of Bern, Bern, Switzerland; 3 CTU Bern, University of Bern, Bern, Switzerland; 4 Queensland Centre for Population Research, School of Earth and Environmental Sciences, The University of Queensland, Brisbane, Australia; 5 Institute of Primary Health Care (BIHAM), University of Bern, Bern, Switzerland; 6 Department of Epidemiology, Biostatistics, and Occupational Health, McGill University, Montreal, Canada; 7 Population Health Laboratory (#PopHealthLab), University of Fribourg, Fribourg, Switzerland; The University of the South Pacific, FIJI

## Abstract

**Background:**

Compared to other OECD countries, Switzerland has the highest rates of hip (HA) and knee arthroplasty (KA).

**Objective:**

We assessed the regional variation in HA/KA rates and potential determinants of variation in Switzerland.

**Methods:**

We conducted a population-based analysis using discharge data from all Swiss hospitals during 2013–2016. We derived hospital service areas (HSAs) by analyzing patient flows. We calculated age-/sex-standardized procedure rates and measures of variation (the extremal quotient [EQ, highest divided by lowest rate] and the systemic component of variation [SCV]). We estimated the reduction in variance of HA/KA rates across HSAs in multilevel regression models, with incremental adjustment for procedure year, age, sex, language, urbanization, socioeconomic factors, burden of disease, and the number of orthopedic surgeons.

**Results:**

Overall, 69,578 HA and 69,899 KA from 55 HSAs were analyzed. The mean age-/sex-standardized HA rate was 265 (range 179–342) and KA rate was 256 (range 186–378) per 100,000 persons and increased over time. The EQ was 1.9 for HA and 2.5 for KA. The SCV was 2.0 for HA and 2.2 for KA, indicating a low variation across HSAs. When adjusted for procedure year and demographic, cultural, and sociodemographic factors, the models explained 75% of the variance in HA and 63% in KA across Swiss HSAs.

**Conclusion:**

Switzerland has high HA/KA rates with a modest regional variation, suggesting that the threshold to perform HA/KA may be uniformly low across regions. One third of the variation remained unexplained and may, at least in part, represent differing physician beliefs and attitudes towards joint arthroplasty.

## Introduction

Osteoarthritis (OA) of the weight bearing joints, including the hip and knee, are major contributors to chronic pain and pain-related disability [1, 2]. In 2010, the global age-standardised prevalence of symptomatic radiographically confirmed hip OA was estimated to be 0.85% and 3.8% for knee OA [2]. Treatment guidelines recommend non-surgical treatments, such as pain medications, exercise, and physical therapy in early disease [3–5]. Surgery is recommended in selected patients with function limiting pain despite non-surgical treatment [4, 6]. The benefits of total hip arthroplasty (HA) and knee arthroplasty (KA) include pain relief and improved function [3, 6]. Both procedures have been shown to be cost-effective [7–9].

Despite the overall success of HA and KA, several factors need to be considered before surgery is recommended. While the 30-day procedure-related complication rate is low (usually below 5–6%) [10–12], not every patient will benefit from a total joint arthroplasty, with 7–23% of patients suffering from pain after HA and 10–34% after KA and 2.5% developing joint infection [8, 11]. Further, the risk of prosthetic failure increases when the surgery is performed earlier in life because the prosthetic joint is used more intensively and longer [13]. Various factors have been found to influence HA and KA rates, including patient and physician preferences [14–16].

**Fig 1:**
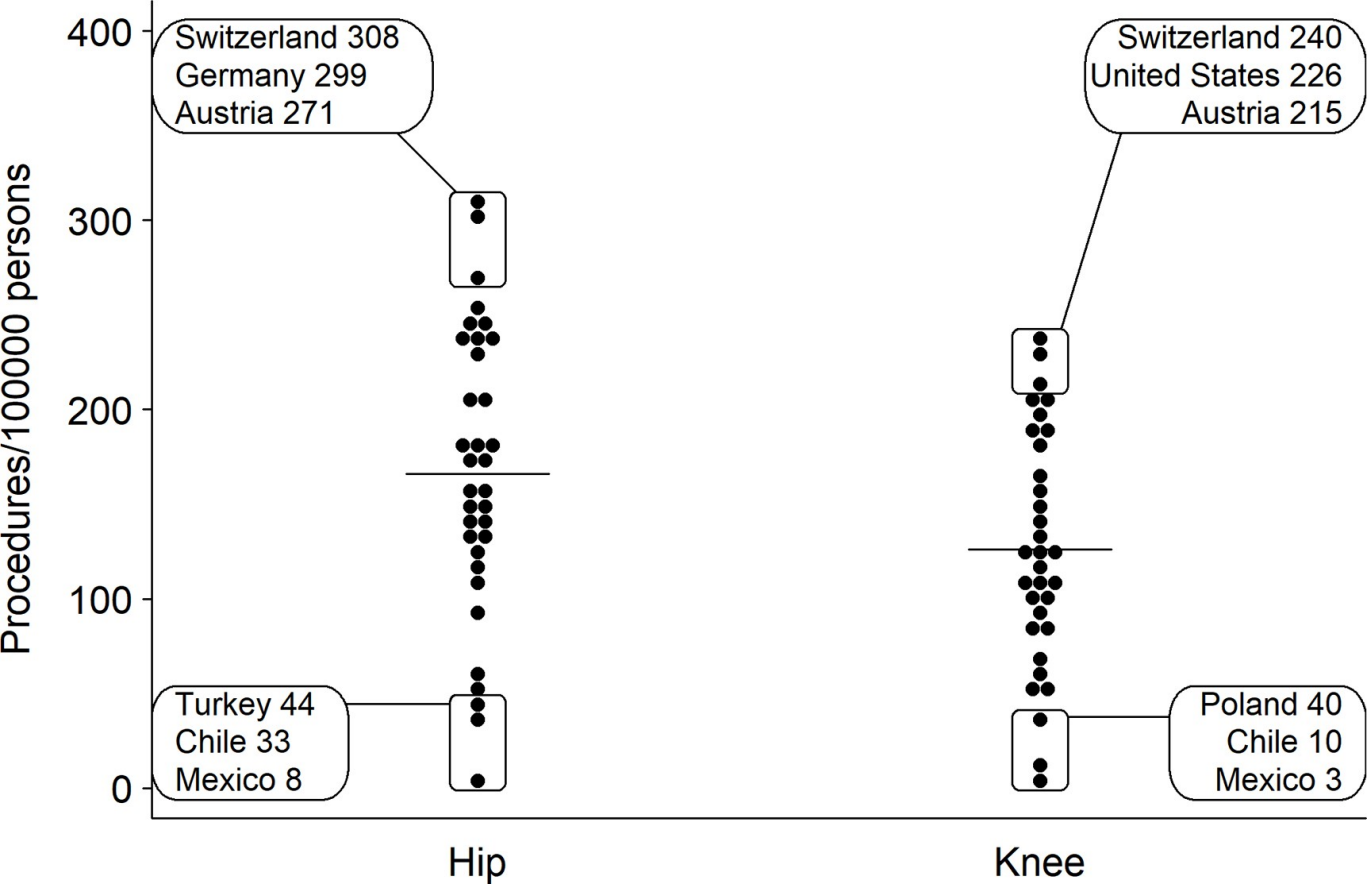
Comparison of age-standardized hip and knee arthroplasty rates across OECD countries in 2015 [17]. Horizontal line represents the OECD average rate.

On average, the rate of HA increased by 30% and the rate of KA by 50% in OECD countries between 2000 and 2015 and the procedure rate varied widely across countries [17]. Switzerland, a country with universal health care coverage, had the highest rate of HA/KA of all OECD countries in 2015 (**Fig 1**) [17]. With 308 HAs and 240 KAs per 100,000 persons, the Swiss procedure rates were almost twice as high as the OECD average [17]. While variations in the use of elective procedures can be partially attributed to differences in healthcare systems and culture [18, 19], the high Swiss HA/KA procedure rates relative to other highly developed OECD countries remain poorly understood. To increase our understanding of which determinants drive the use of HA/KA, we examined regional variations in Switzerland and explored the influence of potential demographic, cultural, socioeconomic, medical, and supply factors on variation.

## Methods

### Data sources

We conducted a population-based, retrospective, cross-sectional small area variation analysis based on routinely collected patient discharge data from all Swiss non-psychiatric acute care hospitals and Swiss census data for calendar years 2013–2016 [20]. Swiss hospitals are legally obligated to provide the Swiss Federal Statistical Office (SFSO) with an anonymized, standardized data set for each hospital discharge. These data are centrally stored in the Swiss Hospital Discharge Masterfile hosted at the SFSO. Recorded variables include patient age, sex, nationality, insurance type, up to 100 procedure codes based on the Swiss Classification of Operations (CHOP, an adaptation of the U.S. ICD-9-CM volume 3 procedure classification [21]), up to 50 diagnostic codes based on the International Classification of Diseases, 10^th^ revision, German Modification (ICD-10-GM), and the type of admission (elective vs. emergency). Further, the area of patient residence and hospital location within one of 705 Swiss MedStat regions are recorded. MedStat regions represent Swiss census regions based on aggregated ZIP-codes [22]. The SFSO reviews data integrity and completeness by means of a specifically designed software [23]. As hospital reimbursement is directly based on the documentation of the main procedures, data completeness and accuracy of CHOP codes is likely to be very high [24].

We used Swiss National Cohort data [25] and data from the SFSO spatial planning [26] from 2014 to determine the level of urbanization (urban area, peri-urban area, and rural area) and the language (German, French, or Italian) for each MedStat region. We abstracted indicators of socioeconomic status (neighborhood information on rent, education, occupation, and crowding) from 2000 Swiss census data [27]. Finally, we obtained the number of orthopedic surgeons per MedStat region for calendar year 2014 from the Swiss Medical Association. Because our study was based on anonymized administrative data only, it was exempted from ethics committee approval according to the Swiss Human Research Act.

### Derivation of Swiss hospital service areas

Switzerland has compulsory basic health insurance coverage, with voluntary semiprivate and private insurance plans covering additional medical services. The Swiss health care system is highly decentralized. Although Swiss hospital care is primarily organized based on 26 administrative regions (the cantons) that are responsible for health care planning, patients may utilize hospital services outside their canton of residence and the use of cantons as a unit of observation may thus skew procedure rates. We therefore developed a novel, fully automated method to generate reproducible general HSAs using all patient discharge data from the calendar years 2013–2016 [28]. In a first step, we identified 4,105,885 patient discharges aged ≥18 years from 155 Swiss hospitals during calendar years 2013–2016 (**S1 Fig**). Only patients living in Switzerland were considered. We then identified MedStat regions with ≥1 hospital and analyzed patient flows from neighboring MedStat regions. MedStat regions that had the highest proportion of their residents discharged from the same hospital MedStat region were assigned to the same HSA (plurality rule) [29]. HSAs with <50% of the patients being treated within the same HSA or <10 discharges overall were merged with the neighboring HSA which received most discharges until >50% and ≥10 discharges occurred within each HSA. This process yielded 63 general HSAs. In a second step, we identified patient discharges with procedure-specific CHOP codes for a first-time HA (CHOP codes total HA 81.51 and partial HA 81.52) or KA (81.54) from all Swiss acute care hospitals during calendar years 2013–2016 using the Swiss Hospital Discharge Masterfile. As HA and KA are procedures that are not performed in every hospital, we analyzed patient flows for HA and KA. Using the procedure described above, HSAs were further aggregated into 55 intervention-specific HSAs. We then drew visual maps of the 55 final HSAs using Geographical Information System (GIS)-compatible vector files.

### Study population

Overall, we identified 92,557 adult discharges with specific codes for HA and 71,134 adult discharges with specific codes for KA who had a MedStat region code. We excluded all discharges related to emergency procedures (hip n = 17,360, knee n = 412, **S1 Fig**), fractures (hip fracture S72.0, 72.1, 72.2; knee fractures S82.1, S72.4; tumor related fractures M90.75, M90.76; n = 1722 for HA and n = 227 for KA) and accidents (vehicle accidents (ICD 10 codes V01-X59), self-harm (X60-X84), and physical assaults (X85-Y09); n = 507 for HA and n = 472 for KA), and revision surgery (HA n = 445, KA n = 124), leaving a final study population of 69,578 patient discharges for HA and 69,899 patient discharges for KA.

### Measures of variation

We calculated age- and sex-standardized HA and KA procedure rates per 100,000 persons for each HSA using procedure counts and 2013–2016 census data for the adult Swiss population [30]. We used direct standardization with 5-year age bands (18 to 19, 20 to <25, […] to ≥95 years). As the prevalence of OA is highest in the elderly population and to increase comparability with prior studies [31, 32], we also calculated unadjusted and age-/sex-standardized rates of HA and KA in persons aged ≥65 years. To examine the variation in procedure rates across Swiss HSAs, we determined the extremal quotient (EQ), which is the highest divided by the lowest procedure rate. While the EQ is an intuitive measure of variation, it is prone to distortion by extreme values [33]. We also calculated the coefficient of variation (CV), i.e., the ratio of the standard deviation of the procedures rates to the mean rate, the systematic component of variation (SCV), and the Empirical Bayes (EB) statistic [33–35]. Although less intuitive than the EQ, the SCV represents the non-random part of the variation in procedure rates while reducing the effect of extreme values [33, 34, 36]. The SCV is derived from a model that recognizes the differences in rates across areas and the random variation within each area’s true rate. A SCV of >5 is considered indicative of a high variation and an SCV of >10 of a very high variation [33, 36]. The EB statistic is another measure of the non-random part of the variation using the Penalized Quasi Likelihood method which is based on the assumption that the log-relative risks are normally and identically distributed [35]. The EB statistics is not influenced by procedure rates and measures the non-random variation, with a result of zero indicating no variation across HSAs [35].

### Determinants of variation

Because differences in illness incidences and socioeconomic factors are possible and legitimate causes of variation [33], the following potential determinants of warranted (need-based) variation at the HSA- level were explored: demographics (age, sex), language area, urbanization, socioeconomic factors (socioeconomic position, additional semiprivate/private insurance, Swiss citizenship), population health (burden of disease), and supply factors (number of orthopedic surgeons). We used the Degree of Urbanization (DEGURBA) definition by the European statistical office [37, 38] (large urban area, small urban area, and rural area) to assign the level of urbanization with the most inhabitants for each HSA. The same approach was used for language (German vs. French/Italian). We used three measures to assess socioeconomic factors that may influence access to care and HA/KA procedure rates: the Swiss Neighborhood Index of Socioeconomic Position (SSEP), insurance status, and Swiss citizenship. The SSEP consists of four domains (median rent per m^2^, proportion of households led by a person with no/low education, proportion headed by a person in manual/unskilled occupation, and mean crowding, all on the neighborhood level). The SSEP of each HSA was calculated using the mean value of the SSEP of MedStat regions within a HSA [27]. The SSEP varies between 0 (worst) and 100 (best) and correlates well with mortality [39]. Although all Swiss residents are legally obliged to buy a general health insurance, only a minority can afford to purchase additional semiprivate/private insurance [40], which entitles to receive a better hotel service in the hospital and the services of a more senior physician. There is an ongoing debate in Switzerland whether semiprivate/private insurance coverage (which results in a higher physician reimbursement) drives procedure rates [41]. Swiss nationals tend to be wealthier, better educated, and have a better language proficiency and health literacy than the (often) poorer and younger immigrant population [42]. We determined the percentage of discharges with semiprivate/private vs. general health insurance and Swiss vs. foreign citizenship for each HSA. The proportion of discharges with a semiprivate/private insurance and a Swiss citizenship were extrapolated to the entire population of a given HSA. As a proxy for the population burden of disease, we calculated age and sex-standardized incidence rates of hip fracture (ICD 10 codes S720, S721, S722), cancer of the colon (ICD 10 codes C18, C19 and CHOP codes 457, 458 or 46) and lung treated surgically (ICD 10 codes C34 and CHOP codes 323, 324, 325, 326, or 329), acute myocardial infarction (ICD 10 codes I21), or stroke (ICD 10 codes I63, I64) for each HSA [43]. These conditions usually require inpatient care and have a very small regional variation [44]. Thus, differences in these conditions are likely to reflect true regional differences in burden of disease rather than differences in coding intensity or supply factors [43, 44].

To explore determinants of HA/KA rates in Switzerland, we used progressively adjusted multilevel Poisson regression to model the procedure rates in each HSA using age- and sex-strata. Age was divided into 5-year strata. Model 1 included only the calendar year of the procedure. Model 2 was additionally adjusted for patient age and sex. Model 3 was additionally adjusted for HSA-level language, urbanization, and socioeconomic factors (SSEP, insurance status, Swiss citizenship). Model 4 was further adjusted for HSA-level burden of disease (the sum of the age-/sex-adjusted incidence rates per 1000 persons for hip fracture, cancer of the colon or lung treated surgically, acute myocardial infarction, or stroke). Model 5 was additionally adjusted for the number of orthopedic surgeons per HSA. We presented graphically the variation in HSA rates as average predicted HA/KA rates per 100,000 persons per HSA derived from the multilevel regression models.

We expressed the impact of determinants on HA/KA rates as incidence rate ratios (IRRs), defined as the HA/KA rate in the defined category (e.g., women) relative to the estimated HA/KA rate in the reference category (e.g., men). We also determined the percentage reduction in procedure variation across the 55 HSAs by examining the variance of the random intercept. We considered the residual, unexplained variation of the fully adjusted model a proxy for unwarranted variation, i.e. the variation that cannot be attributed to patient need [33, 45–48]. Statistical analyses were performed using Stata version 15.1 (StataCorp, College Station, TX, USA) and R statistical software version 3.4.2 [49].

## Results

### Characteristics of Swiss HSAs and the study population

The median population size per HSA was 329,642 persons (interquartile range [IQR] 175,060–697,290), with a median population density of 938 persons per km^2^ (IQR 292–1,693), a mean SSEP of 62 points (standard deviation (SD) 6), and mean number of orthopedic surgeons per HSA of 41 (SD 74.0, **S2 Fig**). Thirty-nine HSAs were located in the Swiss German-speaking and 16 in the French- and Italian-speaking regions. Patients who underwent HA or KA had mean age of 68 years (SD 11) and 69 years (SD 10), respectively. The majority of patients undergoing joint arthroplasty were women (HA 51%, KA 60%) and Swiss nationals (HA 93%, KA 91%), and most had general insurance coverage (HA 66%, KA 67%; **Table 1**).

**Table 1:** Characteristics of the study population undergoing hip (69,578) or knee (N = 69,899) arthroplasty during calendar years 2013–2016.

Characteristics	Hip arthroplasty	Knee arthroplasty
	n (%)	
Age		
18–49	4796 (7)	2284 (4)
50–59	11,366 (16)	11349 (16)
60–69	20,727 (30)	23357 (33)
70–79	22,482 (32)	23860 (34)
≥80	10,207 (15)	9049 (13)
Gender		
Male	33,855 (49)	28,029 (40)
Female	35,723 (51)	41,870 (60)
Insurance class		
General	45,922 (66)	46554 (67)
Semi-private	15,964 (23)	15783 (23)
Private	7691 (11)	7562 (11)
Citizenship		
Swiss	64725 (93)	63400 (91)
Non-Swiss	4853 (7)	6499 (9)

### Variation in procedure rates across HSAs

**Fig 2:**
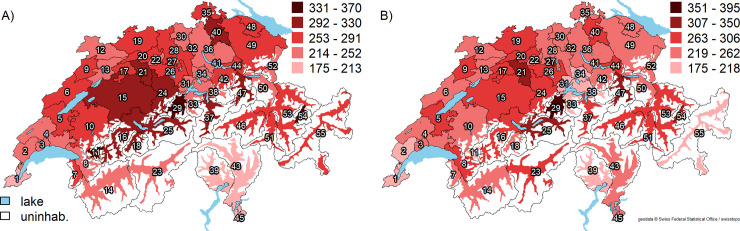
Age- and sex-standardized hip and knee arthroplasty procedure rates per 100,000 persons across 55 Swiss hospital service areas (average rate for calendar years 2013–2016). Panel A: Hip arthroplasty. Panel B: Knee arthroplasty. Abbreviations: uninhab. = uninhabited area. Average age- and sex standardized HA/KA rates for each HSA are shown as red-scale categories per 100,000 persons. Reprinted from the Federal Office of Topography swisstopo, Switzerland https://shop.swisstopo.admin.ch/en/products/maps/overview/relief and shape files derived from postcode-level shape file used to create map of Switzerland, e.g., https://www.geocat.ch/geonetwork/srv/ger/md.viewer#/full_view/973cd117-f1ed-481) under a CC BY license, with permission from Alexandra Frank, original copyright 2006.

The mean age- and sex-standardized HA rate was 263 (range 176–339) per 100,000 persons (**Fig 2**, Panel A). The EQ was 1.9, the CV 0.17, the EB 0.02, and the SCV 1.9, indicating a low variation across HSAs. In patients aged ≥65 years, the mean age-/sex-standardized HA rate was 755 procedures (range 555–1096) per 100,000 persons. The mean age- and sex-standardized KA rate was 255 (range 185–387) per 100,000 persons (**Fig 2**, Panel B). As for HA, the variation in KA rates was low across HSAs (EQ 2.1, CV 0.18, EB 0.02, and SCV 2.6). In patients aged ≥65 years, the mean age- and sex-standardized KA rate was 760 (range 446–1193) per 100,000 persons. Detailed age- and sex-standardized HA and KA procedure rates for each HSA are shown in the **S1 Table.**

**Fig 3:**
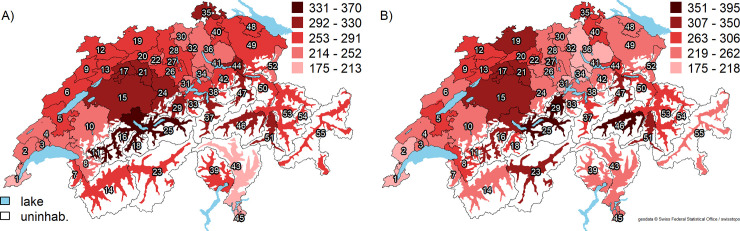
Average predicted hip and knee arthroplasty across 55 Swiss hospital service areas derived from models with progressive adjustment. Panel A: Hip arthroplasty. Panel B: Knee arthroplasty. Abbreviations: uninhab. = uninhabited area; HSA = hospital service area. Average predicted HA/KA rates for each HSA are shown as red-scale categories per 100,000 persons. Adjusted for the year of the intervention, age and sex, language region, level of urbanization, the median Swiss Neighborhood Index of Socioeconomic Position, insurance status, Swiss citizenship, population health, and the number of orthopedic surgeons. Reprinted from the Federal Office of Topography swisstopo, Switzerland https://shop.swisstopo.admin.ch/en/products/maps/overview/relief and shape files derived from postcode-level shape file used to create map of Switzerland, e.g., https://www.geocat.ch/geonetwork/srv/ger/md.viewer#/full_view/973cd117-f1ed-481) under a CC BY license, with permission from Alexandra Frank, original copyright 2006.

After full adjustment for demographics, cultural and socioeconomic factors, burden of disease, and number of orthopedic surgeons, the predicted HA rates varied between 175 and 366 per 100,000 persons (**Fig 3**, Panel A), of which four were above 330 per 100,000 persons (HSA number 11, 16, 18, and 25, all located in central Switzerland) and two were below 200 per 100,000 persons (HSAs 1 and 43). For KA, the highest predicted procedure rates (351–395 per 100,000 persons) were found in three HSAs (number 25, 29, and 46) and the lowest predicted procedure rates (175–219 per 100,000 persons) in six HSAs (number 1, 2, 31, 32, 34, and 36) (**Fig 3**, Panel B).

### Determinants of variation in procedure rates

During 2013 to 2016 procedure rates increased by 5% for HA (IRR 1.05; 95% CI 1.02–1.08) and 8% for KA (IRR 1.08; 95% CI 1.05–1.11). Patient demographics, language region, and socioeconomic factors were the main determinants of procedure variation across HSAs in HA (**Table 2**) and KA (**Table 3**). For HA, age was associated with higher procedure rates (IRR 5.3 [95% CI, 5.1–5.5) for persons aged 75 - <80 years compared to persons aged 50 - <55years), whereas women had an 8% lower procedure rate than men (IRR 0.92; 95% CI, 0.90–0.93). Swiss citizenship was associated with an 11% higher procedure rate than foreign citizenship (IRR 1.11; 95% CI, 1.06–1.17). Language region, urbanization, SSEP, semiprivate/private insurance coverage, burden of disease, and the number of orthopedic surgeons were not significantly associated with procedure rates. Compared to the year-adjusted model, adjustment for age/sex resulted in a 29.9% reduction in the variance of HA rates and further adjustment for cultural/socioeconomic factors resulted in an additional 45% reduction in variance. Adjustment for burden of disease and the number of orthopedic surgeons did not decrease the variance (total variance explained by the full model 74.7%).

**Table 2:** Determinants of variance in the incidence rates of hip arthroplasty across Swiss HSAs.

		Model 1*	Model 2†	Model 3‡	Model 4#	Model 5&
		Incidence rate ratio (95% CI)§				
**Case year**	2013	Reference	Reference	Reference	Reference	Reference
	2014	1.01 (0.98–1.03)	1.00 (0.98–1.02)	1.00 (0.98–1.02)	1.00 (0.98–1.02)	1.00 (0.98–1.02)
	2015	**1.03 (1.01–1.05)**	1.01 (0.99–1.03)	1.01 (0.99–1.03)	1.01 (0.99–1.04)	1.01 (0.99–1.04)
	2016	**1.07 (1.05–1.09)**	**1.04 (1.02–1.06)**	**1.05 (1.02–1.07)**	**1.05 (1.02–1.08)**	**1.05 (1.02–1.08)**
**Age**	18–49		**0.19 (0.18–0.19)**	**0.19 (0.18–0.19)**	**0.19 (0.18–0.19)**	**0.19 (0.18–0.19)**
	50–54		Reference	Reference	Reference	Reference
	55–59		**1.72 (1.66–1.78)**	**1.72 (1.66–1.78)**	**1.72 (1.66–1.78)**	**1.72 (1.66–1.78)**
	60–64		**2.72 (2.63–2.82)**	**2.72 (2.63–2.82)**	**2.72 (2.63–2.82)**	**2.72 (2.63–2.82)**
	65–69		**3.80 (3.68–3.94)**	**3.80 (3.68–3.94)**	**3.80 (3.68–3.94)**	**3.80 (3.68–3.94)**
	70–74		**4.79 (4.63–4.95)**	**4.79 (4.63–4.95)**	**4.79 (4.63–4.95)**	**4.79 (4.63–4.95)**
	75–79		**5.27 (5.09–5.46)**	**5.27 (5.09–5.46)**	**5.27 (5.09–5.46)**	**5.27 (5.09–5.46)**
	≥80		**3.52 (3.40–3.65)**	**3.52 (3.40–3.65)**	**3.52 (3.40–3.65)**	**3.52 (3.40–3.65)**
**Sex**	Male		Reference	Reference	Reference	Reference
	Female		**0.92 (0.90–0.93)**	**0.92 (0.90–0.93)**	**0.92 (0.90–0.93)**	**0.92 (0.90–0.93)**
Language region	German			Reference	Reference	Reference
	French/Italian			0.94 (0.88–1.00)	0.94 (0.88–1.00)	0.94 (0.88–1.00)
Degree of urbanization	Urban			Reference	Reference	Reference
	Peri-urban			1.04 (0.97–1.11)	1.04 (0.97–1.12)	1.03 (0.95–1.13)
	Rural			1.06 (0.96–1.18)	1.07 (0.96–1.18)	1.06 (0.94–1.19)
Mean SSEP (per 10 units)				1.07 (0.98–1.17)	1.08 (0.98–1.17)	1.08 (0.99–1.18)
(Semi)private insurance (per 10% change)				0.95 (0.90–1.00)	0.95 (0.90–1.00)	0.95 (0.90–1.00)
**Swiss citizenship (per 10% change)**				**1.11 (1.06–1.17)**	**1.11 (1.06–1.17)**	**1.11 (1.06–1.17)**
Burden of disease (per comorbidity/1000 persons)т					0.98 (0.89–1.07)	0.98 (0.89–1.07)
Orthopedic surgeons (per 10 change)**						1.00 (1.00–1.00)
**Remaining variance from the model (%)**^II^			**70.1**	**25.6**	**25.5**	**25.3**

Abbreviations: CI = confidence interval; SSEP = Swiss Neighborhood Index of socioeconomic position.

*Model 1: adjusted for the year of the procedure.

†Model 2: additional adjustment for age and sex.

‡Model 3: additional adjustment for language region, level of urbanization, and regional socioeconomic factors (SSEP, insurance status, and Swiss citizenship).

#Model 4: additional adjustment for regional burden of disease.

&Model 5: additional adjustment for the number of orthopedic surgeons.

§Hip replacement rate in the defined category relative to the hip arthroplasty rate in the reference category. For instance, an incidence rate ratio of 0.95 indicates a 5% lower hip arthroplasty rate in women than in men.

тBurden of disease represents the sum of age-standardized incidence rates for the following comorbidities: hip fracture, colon or lung cancer treated surgically, acute myocardial infarction, and stroke. The IRR is the increase (decrease) in procedure rates when the regional burden of disease increases by 1 comorbidity per 1000 person

**The IRR is the increase (decrease) in procedure rates when the number of orthopedic surgeons increases by 10 orthopedic surgeons.

^II^Expresses the variance in hip arthroplasty rates from the national mean.

**Table 3:** Determinants of variance in the incidence rates of knee arthroplasty across Swiss HSAs.

		Model 1*	Model 2†	Model 3‡	Model 4#	Model 5&
		Incidence rate ratio (95% CI)§				
**Case year**	2013	Reference	Reference	Reference	Reference	Reference
	2014	1.02 (1.00–1.04)	1.01 (0.99–1.03)	1.01 (0.99–1.03)	1.01 (0.99–1.03)	1.01 (0.99–1.03)
	2015	**1.05 (1.03–1.07)**	**1.03 (1.01–1.05)**	**1.02 (1.00–1.05)**	**1.03 (1.01–1.05)**	**1.03 (1.01–1.05)**
	2016	**1.11 (1.09–1.13)**	**1.09 (1.06–1.10)**	**1.07 (1.05–1.10)**	**1.08 (1.05–1.11)**	**1.08 (1.05–1.11)**
**Age**	18–49		**0.10 (0.09–0.10)**	**0.10 (0.09–0.10)**	**0.10 (0.09–0.10)**	**0.10 (0.09–0.10)**
	50–54		Reference	Reference	Reference	Reference
	55–59		**2.06 (1.98–2.14)**	**2.06 (1.98–2.14)**	**2.06 (1.98–2.14)**	**2.06 (1.98–2.14)**
	60–64		**3.60 (3.47–3.73)**	**3.60 (3.47–3.73)**	**3.60 (3.47–3.73)**	**3.60 (3.47–3.73)**
	65–69		**4.57 (4.41–4.73)**	**4.57 (4.41–4.73)**	**4.57 (4.41–4.73)**	**4.57 (4.41–4.73)**
	70–74		**5.59 (5.39–5.79)**	**5.59 (5.39–5.79)**	**5.59 (5.39–5.79)**	**5.59 (5.39–5.79)**
	75–79		**6.14 (5.93–6.37)**	**6.14 (5.92–6.37)**	**6.14 (5.92–6.37)**	**6.14 (5.92–6.37)**
	≥80		**3.32 (3.20–3.44)**	**3.32 (3.20–3.44)**	**3.32 (3.20–3.44)**	**3.32 (3.20–3.44)**
**Sex**	Male		Reference	Reference	Reference	Reference
	Female		**1.31 (1.29–1.33)**	**1.31 (1.29–1.33)**	**1.31 (1.29–1.33)**	**1.31 (1.29–1.33)**
**Language region**	Germanic			Reference	Reference	Reference
	French/Italian			**0.87 (0.81–0.94)**	**0.88 (0.81–0.95)**	**0.88 (0.81–0.95)**
Degree of urbanization	Urban			Reference	Reference	Reference
	Peri-urban			1.00 (0.91–1.09)	1.00 (0.91–1.10)	1.01 (0.90–1.13)
	Rural			1.04 (0.91–1.19)	1.04 (0.91–1.19)	1.04 (0.90–1.21)
Mean SSEP (per 10 points)				1.04 (0.93–1.17)	1.04 (0.93–1.17)	1.04 (0.93–1.17)
**(Semi)private insurance (per 10% change)**				**0.90 (0.85–0.95)**	**0.89 (0.84–0.95)**	**0.89 (0.84–0.95)**
Swiss citizenship (per 10% change)				1.03 (0.97–1.08)	1.02 (0.97–1.08)	1.02 (0.97–1.08)
Burden of disease (per comorbidity/1000 persons)т					0.95 (0.86–1.05)	0.95 (0.86–1.05)
Orthopedic surgeons (per 10 change)**						1.00 (0.99–1.01)
**Remaining variance from the model (%)**II			**74.8**	**36.4**	**36.9**	**37.0**

Abbreviations: CI = confidence interval; SSEP = Swiss Neighborhood Index of socioeconomic position.

*Model 1: adjusted for the year of the intervention.

†Model 2: additional adjustment for age- and sex.

‡Model 3: additional adjustment for language region, level of urbanization, and regional socioeconomic factors (SSEP, insurance status, and Swiss citizenship).

#Model 4: additional adjustment for regional burden of disease.

&Model 5: additional adjustment for the number of orthopedic surgeons.

§Hip arthroplasty rate in the defined category relative to the hip arthroplasty rate in the reference category. For instance, an incidence rate ratio of 0.95 indicates a 5% lower hip arthroplasty rate in women than in men.

тBurden of disease represents the sum of age-standardized incidence rates for the following comorbidities: hip fracture, colon or lung cancer treated surgically, acute myocardial infarction, and stroke. The IRR is the increase (decrease) in procedure rates when the regional burden of disease increases by 1 comorbidity per 1000 person.

**The IRR is the increase (decrease) in procedure rates when the number of orthopedic surgeons increases by 10 orthopedic surgeons.IIExpresses the variance in knee arthroplasty rates from the national mean.

For KA, age was associated with higher procedure rates (IRR 6.14 [95% CI, 5.92–6.37] for persons aged 75 - <80 years compared to persons aged 50 - <50 years) and women had a 31% higher procedure rate than men (IRR 1.31; 95% CI, 1.29–1.33). Residence in a French or Italian-speaking language region was associated with a 12% lower KA rate (IRR 0.88; 95% CI, 0.81–0.95 compared to the German speaking part) and semiprivate/private insurance coverage with a 11% lower procedure rate (IRR 0.89; 95% CI, 0.84–0.95). Urbanization, SSEP, Swiss citizenship, burden of disease, and the number of orthopedic surgeons were not statistically significantly associated with procedure rates. Compared to the year-adjusted model, adjustment for age/sex resulted in a 25.2% reduction in the variance of KA rates and further adjustment for cultural/socioeconomic factors resulted in an additional 37.8% reduction in variance. Further adjustment for burden of disease and the number of orthopedic surgeons resulted in almost no variance decrease (total variance explained by the models 63%).

## Discussion

Our analysis demonstrates that despite a substantial increase in HA and KA rates between 2013 and 2016, the procedure rates for HA and KA were relatively homogenous across 55 Swiss HSAs. While demographic factors (age, sex), language, and socioeconomic factors (insurance status, Swiss citizenship) were influential, procedure rates were not associated with the number of orthopedic surgeons. About 75% of the variation in HA and 63% of the variation in KA procedure rates were explained by differences in age, sex, language, and socioeconomic factors.

The number of HA and KA increased rapidly between 2000 and 2015 in most OECD countries [17]. On average, the rate of HA increased by 30% and the rate of KA nearly doubled [17]. In Switzerland, the OECD country with the highest HA and KA rates in 2015, the increase was 28% for HA and 122% for KA since 2002. Many factors may contribute to the high average Swiss HA/KA rates, such as universal health care coverage with a easy access to care [40], supply factors (e.g., number of available hospital beds [33, 50], and financial incentives. The introduction of the Swiss diagnosis-related groups (DRG) payment system in 2012 could also have resulted in an expansion in procedure volume, as shown in France, a neighboring country of Switzerland [51].

Our data show that although the HA rate increased by 5% and KA rate by 9% in the period between 2013 and 2016, the variation in procedure rates across Swiss HSAs was remarkably low and comparable to Switzerland’s neighboring countries, Germany and France, which had a 2.4-fold and 2.8-fold variation in KA during calendar years 2010/11, respectively [52]. The 1.9-fold variation for HA and a 2.5-fold variation for KA observed in our study contrasts with an earlier Swiss study that showed a 3.0-fold variation for KA and a 3.4-fold variation in HA across Swiss HSAs between 2002 and 2005 [53]. The SCV decreased between 2002 and 2005 and the current study period from 4.5 to 1.9 for HA and from 5.3 to 2.2 for KA [53]. The high and increasing procedure rates coupled with a decreasing regional variation indicate that the threshold to perform HA/KA may be decreasing throughout Switzerland. While HA/KA in selected patients with function-limiting pain despite non-surgical treatment often results in pain relief, improved function, and a better quality of life [3, 4, 6], a substantial proportion of HA/KA procedures is performed in patients earlier in life with milder loss of mobility and symptoms [54, 55]. Using validated clinical appropriateness criteria for HA/KA [56–58], approximately 14% to 20% of HA [56, 57] and 34% [58] to 68% of KA [56] are considered inappropriate. Patient preferences for joint arthroplasty may also vary across areas and could explain in part observed geographical variations [19].

Patients often choose to undergo an elective procedure based on their previous experiences, beliefs and expectations of positive outcomes and may underestimate the potential harms [16, 19, 59]. Total joint arthroplasty earlier in life results in an increased need of revision surgery. Overall, 35% of patients who receive their first joint arthroplasty below the age of 70 years undergo revision surgery compared to 5% of patients who receive their first joint arthroplasty after the age of 70 years [13]. In our study, 52% of patients undergoing HA/KA were aged <70 years and many of those may require revision surgery later in time. Evidence suggests that for preference sensitive procedures, such as HA and KA, shared decision making using a decision aid may improve the decision quality and result in fewer surgeries, without compromising health outcomes [60–62].

As the prevalence of OA increases with age [63], age was the strongest predictor of procedure rates in our study. Although the incidence of hip/knee OA is higher in women [63], women were paradoxically less likely to undergo HA than men. A potential explanation for this observation is the fact that we focused on elective joint arthroplasty and excluded emergency/fracture related HA from our analysis. As two-thirds of osteoporotic hip fractures occur in women [64], the exclusion of emergency HA could have resulted in a lower incidence for HA in women. Although evidence suggests that men and women are equally willing to undergo HA and KA, women are less likely to discuss arthroplasty with their physician and are less likely to undergo both procedures [65].

Microcultural factors may also play a role in how health care is used [59]. Inhabitants of French/Italian speaking regions were shown to have higher per-capita healthcare costs [66] and overuse of medical services [42], to consult specialists more frequently [67], and to consume more health care at the end of life than people in Swiss German speaking areas [68, 69]. The lower joint arthroplasty rates (especially for KA) in French/Italian-speaking areas indicate that the higher health care use in these areas [42, 59, 66–69] may not necessarily extend to invasive orthopedic procedures. Whether differential procedure rates are due to differences in patient preferences, health literacy [70], involvement in shared-decision making [71], or local physicians’ attitudes and practices is unknown and must be further explored.

HSAs with a higher proportion of Swiss citizens were more likely to have HA than those with a lower proportion. A potential explanation is that the immigrant and Swiss citizen population differ in terms of other factors that may increase the risk for OA, such as biomechanical (e.g., joint morphology) and genetic factors, bone mineral density, and obesity [72].

Although a private health insurance is related with higher physician reimbursement in Switzerland and thus could drive procedure rates [66], regions with a higher proportion of privately insured inhabitants had lower joint (especially knee) arthroplasty rates. People who are able to afford private insurance plans may be more health conscious and therefore may have a lower prevalence of hip and knee OA [42]. Urbanicity as a proxy for access to care was not predictive of procedure rates, indicating that Swiss universal health care coverage provides adequate access to care even in remote areas [73].

Somewhat surprisingly, the number of orthopedic surgeons did not influence procedure rates. Several higher-volume areas (e.g., HSA 37 and 47) had comparatively low numbers of orthopedic surgeons, and several low-volume areas had higher numbers (e.g., HSA 1 and 43). These findings indicate that qualitative aspects, such as individual physicians’ beliefs and preferences regarding the efficacy of non-surgical treatments and the indication for HA/KA, may play a more important role than the absolute number of surgeons [74]. Indeed, orthopedic surgeons' opinions or enthusiasm for KA has been found to be the dominant modifiable determinant of area variation [50].

Our work has potential limitations. First, no national data on the prevalence of hip and knee OA was available and we cannot say if variations in procedure rates could be potentially explained by differing regional OA prevalences. However, there is no plausible explanation why the prevalence or severity of OA would vary across geographically close Swiss HSAs. Second, the derivation of Swiss HSAs was based on MedStat regions, which contain one or more hospitals. Thus, a hospital-level analysis was not feasible. Third, to adjust for regional population health (burden of disease), we used the sum of the incidence of hip fractures, colon/lung cancer surgery, acute myocardial infarctions, and strokes for each as a proxy measure and cannot say how precisely this measure estimates the true population health status. Finally, language, SSEP, insurance status, citizenship, and burden of disease represent ecological variables describing the population living in a HSA rather than those receiving HA/KA themselves. Although we found an association between several HSA-level variables (e.g., language) and procedure rates, what is true for a region as a whole may not be necessarily true for individuals within this region (ecological fallacy) [75]. To overcome this potential limitation, individual patient data on the presence of hip/knee osteoarthritis as well as potential determinants would be necessary. Finally, we used Poisson models rather than negative binomial models because the comparison of the residual variances from negative binomial models is difficult due to differing quantities of variance going into the overdispersion parameter. Thus, we cannot exclude the possibility that our estimates, particularly the standard errors, may be too small.

In our study, only about two thirds of the modest procedure variation across Swiss HSAs were explained by differences in demographic, cultural, and socioeconomic factors. The residual variation could be due to regional differences in patient preferences or the prevalence/severity of OA, or local differences in physicians’ attitudes towards performing joint arthroplasty [76]. Evidence suggests that orthopedic surgeons' enthusiasm for KA may be the dominant modifiable determinant of area variation [50]. Finally, the uptake of guideline recommendations for non-surgical options to treat hip and knee OA appears to be poor amongst orthopedic surgeons [74] and effective guideline implementation strategies may be needed to reduce residual, potentially unwarranted variation in HA/KA rates.

In conclusion, Switzerland has high elective HA/KA procedures rates with a relatively modest regional variation, indicating that the threshold to perform HA/KA may be uniformly low. While demographic, linguistic, and socioeconomic factors were associated with procedure rates, the number of orthopedic surgeons was not. About one third of the variation in procedure rates remained unexplained and may, at least in part, represent differing physician beliefs and attitudes towards joint arthroplasty rather than differences in patient need and preferences.

## Supporting information

S1 Checklist STROBE statement. (DOC)

S1 Fig Study flow chart. Abbreviations: HA = hip arthroplasty; K = knee arthroplasty; ICD codes X/Y/Z = ICD-10 codes X60–84 (self-harm), Y09–84 (crime related injuries, complications), and Z00–99 (preventive medicine (e.g. vaccination)).
(PNG)

S2 Fig Number of orthopedic surgeons by hospital service area. Abbreviations: uninhab. = uninhabited area Each circle represents the proportional number of orthopedic surgeons for each HSA. Reprinted from the Federal Office of Topography swisstopo, Switzerland https://shop.swisstopo.admin.ch/en/products/maps/overview/relief and shape files derived from postcode-level shape file used to create map of Switzerland, e.g., https://www.geocat.ch/geonet work/srv/ger/md.viewer#/full_view/973cd117-f1ed-481) under a CC BY license, with permission from Alexandra Frank, original copyright 2006.
(TIFF)

S1 Table Age- and sex-standardized rates per 100’000 persons by hospital service area. (DOCX)
