# Supplementary figures and images for: Regional variation in hip and knee arthroplasty rates in Switzerland: A population-based small area analysis
Source: PLoS One. 2020 Sep 21;15(9):e0238287. doi: 10.1371/journal.pone.0238287 (PMC7505431; doi:10.1371/journal.pone.0238287)

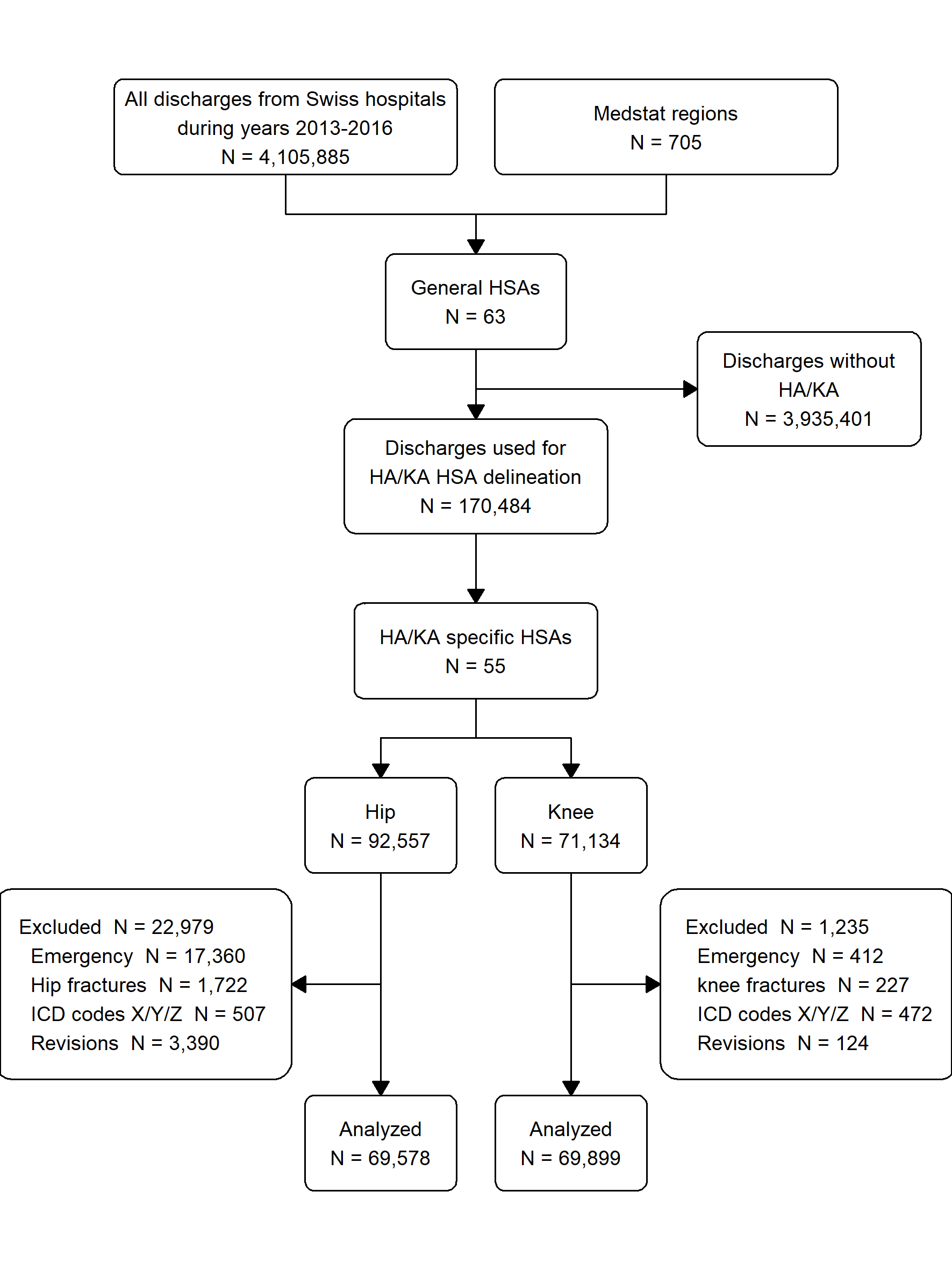

Supplement: S1 Fig — Abbreviations: HA = hip arthroplasty; K = knee arthroplasty; ICD codes X/Y/Z = ICD-10 codes X60–84 (self-harm), Y09–84 (crime related injuries, complications), and Z00–99 (preventive medicine (e.g. vaccination)). (PNG) [file pone.0238287.s002.png]

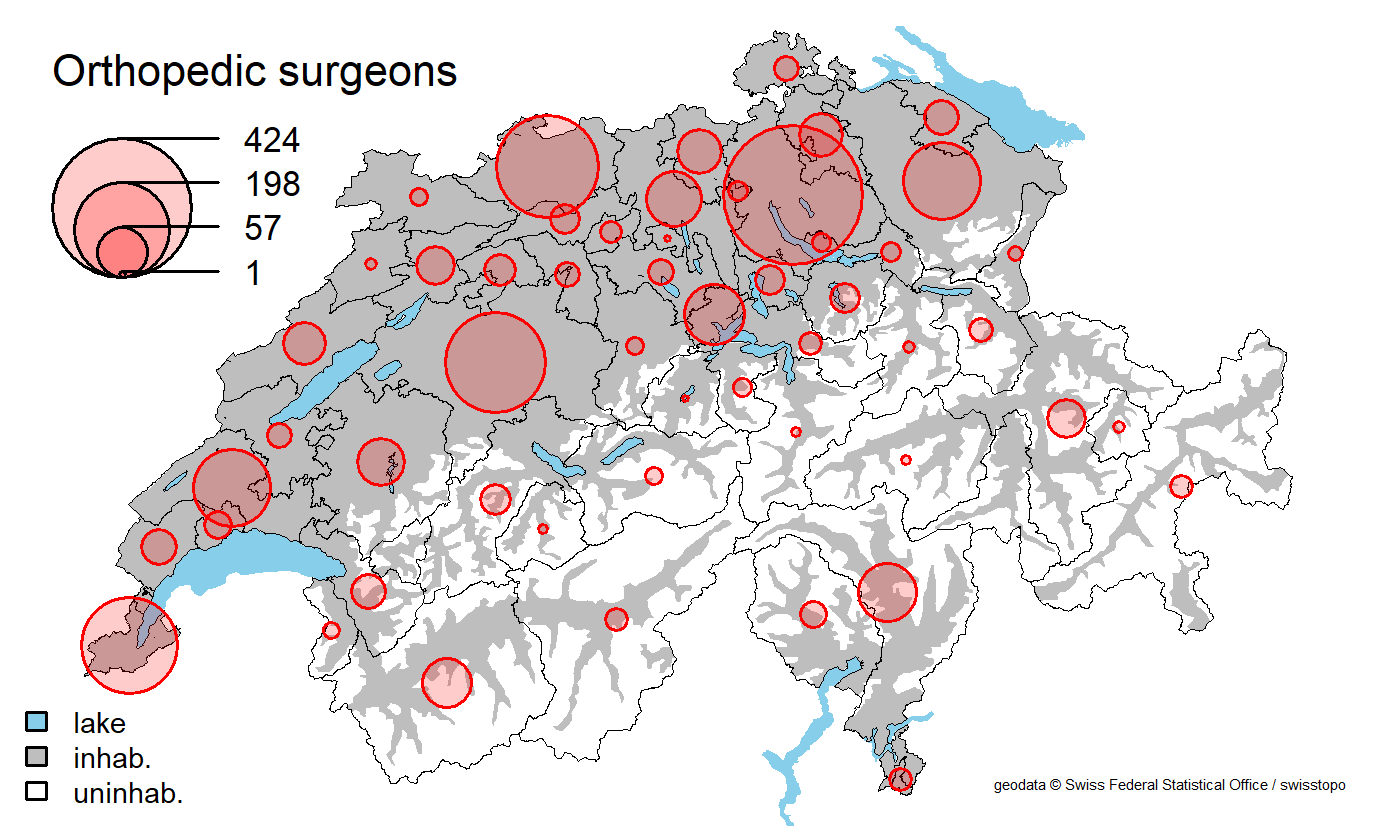

Supplement: S2 Fig — Abbreviations: uninhab. = uninhabited area Each circle represents the proportional number of orthopedic surgeons for each HSA. Reprinted from the Federal Office of Topography swisstopo, Switzerland https://shop.swisstopo.admin.ch/en/products/maps/overview/relief and shape files derived from postcode-level shape file used to create map of Switzerland, e.g., https://www.geocat.ch/geonet work/srv/ger/md.viewer#/full_view/973cd117-f1ed-481) under a CC BY license, with permission from Alexandra Frank, original copyright 2006. (TIFF) [file pone.0238287.s003.tiff]
