# Supplementary material for: Regional variation in hip and knee arthroplasty rates in Switzerland: A population-based small area analysis
Source: PLoS One. 2020 Sep 21;15(9):e0238287. doi: 10.1371/journal.pone.0238287 (PMC7505431; doi:10.1371/journal.pone.0238287)
Supplement: S1 Table — (DOCX) [file pone.0238287.s004.docx]

**S1 Table. Age- and sex-standardized rates per 100’000 persons by hospital service area.**

| **Hip arthroplasty** | | | **Knee arthroplasty** | | |
| --- | --- | --- | --- | --- | --- |
| **HSA** | **All** | **≥65** | **HSA** | **All** | **≥65** |
| 45 | 176.3 | 555.2 | 34 | 185.3 | 584.6 |
| 32 | 185.0 | 626.9 | 1 | 188.3 | 662.8 |
| 43 | 185.7 | 584.0 | 11 | 191.4 | 446.4 |
| 1 | 205.9 | 660.4 | 39 | 191.9 | 600.1 |
| 39 | 212.2 | 603.1 | 2 | 194.5 | 660.6 |
| 12 | 222.5 | 649.3 | 45 | 195.1 | 617.8 |
| 34 | 224.2 | 675.0 | 31 | 208.9 | 705.3 |
| 31 | 226.4 | 697.3 | 33 | 209.6 | 646.2 |
| 36 | 230.5 | 714.4 | 55 | 216.9 | 596.1 |
| 30 | 233.9 | 707.5 | 41 | 220.7 | 691.1 |
| 4 | 236.4 | 732.5 | 14 | 221.1 | 650.8 |
| 8 | 236.6 | 731.2 | 18 | 221.1 | 562.9 |
| 52 | 239.2 | 655.1 | 43 | 222.0 | 687.5 |
| 2 | 239.4 | 778.8 | 3 | 223.3 | 672.9 |
| 13 | 242.0 | 697.8 | 38 | 228.3 | 754.1 |
| 22 | 242.3 | 641.3 | 49 | 229.0 | 654.3 |
| 3 | 244.3 | 792.2 | 36 | 229.2 | 720.7 |
| 14 | 246.9 | 714.2 | 32 | 232.3 | 695.8 |
| 50 | 247.2 | 658.1 | 12 | 233.5 | 698.1 |
| 33 | 249.0 | 687.7 | 30 | 237.8 | 755.8 |
| 38 | 249.8 | 721.2 | 7 | 240.6 | 694.3 |
| 23 | 251.0 | 664.5 | 4 | 242.8 | 768.5 |
| 20 | 251.1 | 714.9 | 42 | 244.2 | 675.1 |
| 55 | 253.3 | 673.6 | 6 | 248.6 | 777.8 |
| 6 | 254.4 | 752.9 | 48 | 248.7 | 742.9 |
| 41 | 254.6 | 785.0 | 13 | 250.5 | 758.0 |
| 19 | 261.4 | 776.0 | 44 | 253.7 | 736.7 |
| 46 | 262.9 | 661.6 | 28 | 255.0 | 783.2 |
| 53 | 262.9 | 762.4 | 35 | 256.2 | 842.0 |
| 10 | 264.9 | 757.9 | 52 | 258.1 | 702.7 |
| 28 | 268.4 | 784.0 | 26 | 260.1 | 787.7 |
| 7 | 268.7 | 809.2 | 10 | 260.2 | 739.8 |
| 9 | 269.4 | 647.1 | 40 | 261.2 | 800.2 |
| 5 | 271.9 | 795.4 | 51 | 261.7 | 695.8 |
| 17 | 273.1 | 764.8 | 22 | 262.5 | 702.2 |
| 48 | 274.5 | 774.1 | 24 | 263.0 | 754.6 |
| 42 | 275.3 | 810.3 | 50 | 265.9 | 813.3 |
| 51 | 276.1 | 791.8 | 20 | 267.9 | 767.3 |
| 49 | 277.3 | 792.3 | 37 | 269.2 | 805.0 |
| 35 | 278.5 | 816.5 | 53 | 269.2 | 795.1 |
| 26 | 281.7 | 801.5 | 17 | 269.2 | 788.9 |
| 27 | 283.9 | 726.2 | 8 | 274.6 | 847.7 |
| 44 | 286.3 | 813.4 | 9 | 280.2 | 794.7 |
| 47 | 290.0 | 860.2 | 23 | 291.1 | 827.2 |
| 37 | 290.7 | 863.2 | 15 | 294.0 | 849.8 |
| 40 | 298.2 | 935.1 | 5 | 295.2 | 874.7 |
| 21 | 302.1 | 802.2 | 16 | 297.5 | 847.1 |
| 15 | 306.0 | 837.8 | 46 | 298.7 | 851.4 |
| 25 | 312.6 | 823.8 | 19 | 300.8 | 927.1 |
| 18 | 325.1 | 779.7 | 47 | 306.1 | 899.8 |
| 24 | 325.4 | 820.8 | 21 | 310.7 | 861.3 |
| 16 | 325.6 | 889.8 | 54 | 317.0 | 1030.0 |
| 11 | 331.0 | 889.9 | 25 | 329.2 | 1004.1 |
| 54 | 338.9 | 1095.5 | 27 | 341.8 | 993.3 |
| 29 | 339.3 | 994.4 | 29 | 387.0 | 1192.9 |

≥65, age-/sex adjusted rates for persons aged ≥65 years
